# Clinical practice variation and need for pediatric-specific treatment guidelines among rheumatologists caring for children with ANCA-associated vasculitis: an international clinician survey

**DOI:** 10.1186/s12969-017-0191-z

**Published:** 2017-08-07

**Authors:** Clara Westwell-Roper, Joanna M. Lubieniecka, Kelly L. Brown, Kimberly A. Morishita, Cherry Mammen, Linda Wagner-Weiner, Eric Yen, Suzanne C. Li, Kathleen M. O’Neil, Sivia K. Lapidus, Paul Brogan, Rolando Cimaz, David A. Cabral, for ARChiVe Investigators Network within the PedVas initiative

**Affiliations:** 1 Clinical Professor, Division of Rheumatology, Department of Pediatrics, University of British Columbia, BC Children’s Hospital, Room K4-119 4480 Oak Street Vancouver, Vancouver, BC V6H 3V4 Canada; 2 Simon Fraser University, Burnaby, BC Canada; 3 University of Chicago Comer Children’s Hospital, Chicago, USA; 4 University of California – Los Angeles, Los Angeles, CA USA; 5 Joseph M. Sanzari Children’s Hospital, Hackensack, NJ USA; 6 Riley Hospital for Children at IU Health, Indianapolis, IN USA; 7 Morristown Medical Center, Morristown, NJ USA; 8 Great Ormond Street Hospital NHS Foundation Trust, London, UK; 9 Ospedale Pediatrico Meyer Firenze, Florence, Italy

**Keywords:** Pediatric rheumatology, Anti-neutrophil cytoplasmic antibody-associated vasculitis, Granulomatosis with polyangiitis, Microscopic polyangiitis, Physician practice patterns, Clinical practice guidelines, Disease classification, Vasculitis treatment, Outcome assessment

## Abstract

**Background:**

Because pediatric antineutrophil cytoplasmic antibody-associated vasculitis is rare, management generally relies on adult data. We assessed treatment practices, uptake of existing clinical assessment tools, and interest in pediatric treatment protocols among rheumatologists caring for children with granulomatosis with polyangiitis (GPA) and microscopic polyangiitis (MPA).

**Methods:**

A needs-assessment survey developed by an international working group of pediatric rheumatologists and two nephrologists was circulated internationally. Data were summarized with descriptive statistics. Pearson’s chi-square tests were used in inferential univariate analyses.

**Results:**

The 209 respondents from 36 countries had collectively seen ~1600 children with GPA/MPA; 144 had seen more than two in the preceding 5 years. Standardized and validated clinical assessment tools to score disease severity, activity, and damage were used by 59, 63, and 36%, respectively; barriers to use included lack of knowledge and limited perceived utility. Therapy varied significantly: use of rituximab rather than cyclophosphamide was more common among respondents from the USA (OR = 2.7 [1.3-5.5], *p* = 0.0190, *n* = 139), those with >5 years of independent practice experience (OR = 3.8 [1.3-12.5], *p =* 0.0279*, n =* 137), and those who had seen >10 children with GPA/MPA in their careers (OR = 4.39 [2.1-9.1], *p =* 0.0011*, n =* 133). Respondents who had treated >10 patients were also more likely to continue maintenance therapy for at least 24 months (OR = 3.0 [1.4-6.4], *p* = 0.0161, *n* = 127). Ninety six percent of respondents believed in a need for pediatric-specific treatment guidelines; 46% supported adaptation of adult guidelines while 69% favoured guidelines providing a limited range of treatment options to allow comparison of effectiveness through a registry.

**Conclusions:**

These data provide a rationale for developing pediatric-specific consensus treatment guidelines for GPA/MPA. While pediatric rheumatologist uptake of existing clinical tools has been limited, guideline uptake may be enhanced if outcomes of consensus-derived treatment options are evaluated within the framework of an international registry.

**Electronic supplementary material:**

The online version of this article (doi:10.1186/s12969-017-0191-z) contains supplementary material, which is available to authorized users.

## Background

Antineutrophil cytoplasmic antibody- (ANCA-) associated vasculitis (AAV) describes the subset of vasculitides primarily involving small vessels: granulomatosis with polyangiitis (GPA, formerly Wegener’s granulomatosis), microscopic polyangiitis (MPA), eosinophilic granulomatosis with polyangiitis (EGPA, formerly Churg-Strauss syndrome), and renal-limited pauci-immune glomerulonephritis [1]. Although rare in childhood, AAV carries a high burden of morbidity. With the use of high-dose corticosteroids and cyclophosphamide, AAV is no longer rapidly fatal in the majority of affected children; however, these treatments have significant adverse effects. Balancing the risks associated with existing therapies against the damage associated with under- or over-treatment remains a challenge. In adult populations, this balance has been incrementally fine-tuned through clinical trials that rely on accurate disease sub-classification and scoring tools to stage disease severity, activity, and damage (Table 1). The rarity of pediatric AAV has limited opportunities for pediatric clinical trials such that management decisions are largely informed by adult data [2], with potential for significant practice variation.

**Table 1:** Definitions of clinical tools included in survey questions

Clinical tool	Description
Birmingham Vasculitis Activity Score (BVAS)	Scoring tool designed to document new or worseningfeatures of clinically active AAV. Items are categorized into 9 groups by organ system [11].
BVAS Version 3	2009 update to BVAS [11, 39].
BVAS for Wegener’s Granulomatosis (WG)	Modification of BVAS that removes features unlikely to occur in GPA [13, 40].
Pediatric Vasculitis Activity Score (PVAS)	Pediatric version of BVAS preliminarily validated in children [12].
EUVAS Severity Score	Classification system for AAV based on disease extent and severity. Subgroups include localized, early systemic, generalized, severe renal, and refractory disease [41].
Five Factor Score	Scoring tool based on factors associated with poor prognosis: cardiac symptoms, gastrointestinal involvement, renal insufficiency, proteinuria, and central nervous system involvement [42].
Wegener’s Granulomatosis Etanercept Trial (WGET) Severity Score	Sub-classification based on modified ACR criteria for limited versus extensive disease [43].
Disease Extent Index (DEI)	Tool for scoring disease activity based on organ system, with distinct domains from those included in BVAS [44].
Physician’s Global Assessment (PGA)	Physician’s global assessment of disease activity on a 10 cm visual analogue scale.
Vasculitis Damage Index (VDI)	Scoring tool used to record damage due to disease or treatment. Items are categorized into 11 groups by organ system with binary scoring [14].
Pediatric VDI (PVDI)	Pediatric modification of the adult VDI; not yet formally validated in children [14, 15].
AAV Index of Damage (AVID)	Tool for grading disease- or treatment-associated damage, with weighting of items based on potential morbidity and mortality [45].
Combined Damage Assessment Index	Combination of items from AVID and VDI, divided into 17 organ-based categories [46].

A 2005 survey of Childhood Arthritis and Rheumatology Research Alliance (CARRA) members elicited overwhelming consensus on the need to study childhood-onset vasculitis independently from adult disease [3]. ARChiVe (A Registry for Childhood Vasculitis) was established in 2007 based on this imperative for pediatric-specific data collection. A concurrent initiative of the Pediatric Rheumatology European Society (PRES) also helped generate time-of-diagnosis data to support the development of pediatric-specific classification criteria and assessment tools. More recently, PedVas (Pediatric Vasculitis Initiative [4]; https://clinicaltrials.gov/ct2/show/NCT02006134) has allowed collection of clinical data and biological samples to ARChiVe. While pediatric clinical trials remain difficult, an international registry such as ARChiVe might provide the opportunity to compare effectiveness of a limited range of standardized treatment options and ultimately generate evidence-based guidelines. This strategy has been pursued for uvenile dermatomyositis, another rare pediatric rheumatic disease [5].

As a first step toward developing treatment guidelines for pediatric AAV, we sought to better understand the diversity of beliefs and practices associated with current care. We conducted an international needs assessment survey of physicians involved in the diagnosis and management of children with GPA and MPA, with the following aims: (1) to assess the level of community experience with pediatric AAV; (2) to assess uptake of existing classification criteria, clinical scoring tools, and treatment guidelines; (3) to assess the extent of variation in current treatment practices; and (4) to determine interest in and capacity for use of pediatric treatment protocols.

## Methods

A survey draft developed by the Pediatric Rheumatology group and a nephrologist at British Columbia Children’s Hospital was finalized with input from the Vasculitis Working Groups of CARRA and PRES. The survey comprised 47 predominantly categorical multi-choice questions in three sections (Additional file 1). The first section addressed practice type and experience with pediatric GPA and MPA (EGPA was excluded given its rarity in pediatrics). Respondents caring for fewer than two children with GPA and/or MPA over the past 5 years were then given the option to exit. The second section queried use and knowledge of classification criteria and formal scoring tools for disease severity, activity and damage (Table 1). Practitioners were also asked about their use of adult treatment guidelines and perceived need for pediatric-specific guidelines. The third section queried motivations for and barriers to participation in clinical registries. Levels of assigned value were requested using a five-point Likert scale from 1 (not important) to 5 (extremely important).

Members of CARRA, the Canadian Alliance of Pediatric Rheumatology Investigators (CAPRI), the Australian Pediatric Rheumatology Group, the PRES-CARRA Vasculitis Working Group, and the Pediatric Rheumatology Bulletin Board (ped-rhe-list@mcmaster.ca) were invited by email to complete the web-based survey (estimated 500 personal email invitations). The survey was administered using REDCap electronic data capture tools [6] hosted at the University of British Columbia (UBC) from July 24 to September 29, 2015. The survey was conducted as a quality assurance initiative for PedVas, which was approved by the Children’s and Women’s Research Ethics Board of UBC (H12-00894). Under Article 2.5 of the Tri Council Policy Statement, quality assurance/improvement activities are not subject to further institutional review.

Descriptive statistics were used to quantify response frequencies and means. Unless otherwise specified, frequencies are reported relative to the total number of respondents completing each question. Odds ratios (OR) were determined as measures of association. Limits of 95% confidence intervals of OR are reported in square brackets. Inferential univariate analysis with two-sided Pearson’s chi-square tests and Benjamini-Hochberg correction for multiple comparisons was used to test 11 hypotheses with a false discovery rate of 5% [7]. Corrected *p*-values are reported. Analysis was performed using Microsoft Excel Version 14.6.7 and Prism Version 5.0a (GraphPad Software Inc., La Jolla, CA).

## Results

### Respondent characteristics

Of 216 respondents opening the survey, 209 completed it, yielding an estimated response rate of approximately 40%. Sixty-five (31%) chose to exit as they had not cared for two or more patients with GPA/MPA in the preceding 5 years. The 144 respondents completing the full survey practiced in 36 countries, predominantly the USA (48%), with others from Canada (13%), Italy (4%), Australia (3%), Germany (4%), Turkey (4%), United Kingdom (4%), Brazil (2%), and Sweden (2%). Most respondents (92%) belonged to one or more national or international rheumatology organizations, including CARRA (56%), PRES (32%), CAPRI (10%), the PRES-CARRA Vasculitis Working Group (10%), and others (18%). Approximately half of respondents had practiced for less than 10 years after formal training (21% for less than 5 years and 26% for 5-10 years); among the rest, 20% each had practiced for 10-20 and 20-30 years and 10% for 30-40 years. The combined lifetime experience of respondents was at least 1600 patients with GPA/MPA (some shared): 47% had seen fewer than ten patients, 39% had seen 10-20, and only 14% had seen more than 20. The majority of respondents (61%) had seen five or fewer patients with a new diagnosis of GPA or MPA within the past 5 years (mode 3, median 5, IQR 3-8 patients).

Most respondents belonged to group practices, sharing diagnostic and treatment decisions for all patients (46%) or managing patients independently while sharing on-call responsibilities (41%). A minority worked in a solo practice (7%) or in another practice arrangement (6%), predominantly hospital-based. For patients who had also been assessed by a nephrologist, the majority of respondents reported making collaborative treatment decisions, 30% in a combined nephrology/rheumatology clinic and 37% in separate clinic settings. Some reported variation in the responsible subspecialty depending on factors such as who had seen the patient first (18%), while 13 and 2% of respondents reported independent management by rheumatology or nephrology, respectively.

### Classification criteria

There were between 129 and 144 responses (89-100%) to subsequent individual questions regarding diagnosis and management. While the majority (73%) reported that they always sub-classify AAV as either GPA or MPA, 26% endorsed only sometimes sub-classifying. Table 2 shows the number of respondents familiar with – and using – existing criteria for defining or classifying AAV. Most were familiar with ACR1990 [8] and EULAR/PRINTO/PRES 2008 [9] classification criteria for GPA, and with CHCC 1994 disease definitions [10]. Serology (cANCA/PR3 versus pANCA/MPO) and EULAR/PRINTO/PRES 2008 criteria [9] were most frequently used for distinguishing between GPA and MPA in clinical practice. Notably, 66% of all respondents reported using more than one set of criteria, and those using informal methods considered histopathology, antibody status, and specific organ involvement. The rationale for sub-classification was predominantly prognostication (73%), influence on treatment choice (48%), involvement in clinical studies/trials (39%), and access to treatments (29%). All respondents felt that distinguishing between GPA and MPA was at least somewhat important for research (mode 4, median 4, IQR 3-4).

**Table 2:** Familiarity with and use of vasculitis classification criteria and disease definitions to differentiate GPA from MPA in children^a^

Criteria	Familiar (%)	Using (%)
EULAR/PRINTO/PRES 2008 criteria [9]	78	57
ANCA (PR3/MPO)	ND^b^	57
ACR 1990 criteria [8, 19]	72	29
CHCC 1994 [10]	62	32
CHCC 2012 [1]	47	
EMA classification algorithm (2007) [22]	18	5
Pediatric Modification of EMA classification algorithm (2012) [21]		
Informal criteria	ND^b^	19
Other formal criteria	ND^b^	1

^a^Data represent percentage of respondents out of *n* = 144 (familiar) or *n* = 143 (using)

^b^No data

### Disease severity, activity, and damage

The proportions of respondents using formal tools to assess disease severity, activity, and damage are shown in Table 3. While most respondents used formal assessment tools for severity (59%) and activity (63%), only 36% had ever formally assessed disease damage. The Birmingham Vasculitis Activity Score (BVAS) [11], the Pediatric Vasculitis Activity Score (PVAS) [12], and BVAS for Wegener’s Granulomatosis [13] – tools designed primarily for assessment of disease activity – were frequently used to stage severity, but tools such as the Five Factor Score and the EUVAS and WGET severity scores were used by a minority. PVAS, BVAS, and physician’s global assessment on a 10 cm visual analogue scale (PGA) were the primary tools used by respondents for scoring disease activity, typically at the time of diagnosis: 39% at only some follow-up visits, 33% at all visits, 16% at prescribed times according to research protocols, and 7% at the time of diagnosis only. Most respondents felt formal assessment of disease activity was somewhat important to clinical management (mode 3, median 5, IQR 3-4), and more important to research (mode 5, median 5, IQR 4-5). Most commonly used tools for scoring disease damage include the Vasculitis Damage Index (VDI) [14] or its pediatric adaptation, PVDI [15]. Similar to scoring of disease activity, respondents assessed disease damage primarily at the time of diagnosis: 35% at only some follow-up visits, 23% at prescribed times only, and 31% at every visit. Respondent rationales for not using formal assessment tools are shown in Table 4 and included use of histopathology, lack of effect on management, lack of familiarity, and inconvenience.

**Table 3:** Use of formal disease severity, activity, and damage scoring tools by survey respondents in the assessment of children with GPA or MPA^a^

Parameter	Scoring tool	Respondents (%)
Severity^b^	PVAS [12]	41
	BVAS [11]	20
	BVAS for WG [13]	19
	EUVAS Severity Score [41]	11
	Five Factor Score [42]	8
	WGET Severity Score [43]	7
	Disease Extent Index [44]	1
	Other	1
	Never formally assess	41
Activity^c^	PVAS [12]	38
	Physician’s Global Assessment	29
	BVAS [11]	19
	BVAS for WG [13]	13
	BVAS version 3 [39]	6
	Other tool	0
	Never formally assess	37
Damage^c^	Pediatric VDI [15]	31
	VDI [14]	10
	AAV Index of Damage [45]	3
	Combined Damage Assessment Index [46]	1
	Other	0
	Never formally assess	64

^a^Data represent percentage of respondents out of ^b^ *n* = 142, ^c^ *n* = 141

**Table 4:** Reported rationales against use of formal assessment tools for scoring disease severity, activity, and damage in the assessment of children with GPA or MPA^a^

Parameter	Rationale against use of tool	Respondents (%)
Severity^b^	Use of histopathological findings	53
	No effect on management	34
	Lack of familiarity with tools	26
	No value added beyond clinical judgment	24
	Inconvenience	16
	Other	5.3
Activity^c^	Lack of familiarity with tools	47
	Inconvenience	43
	No effect on management	22
	Lack of applicability of tools for adults	20
	No value added beyond clinical judgment	18
Damage^d^	Lack of familiarity with tools	58
	Inconvenience	32
	Lack of applicability of tools for adults	20
	No effect on management	18
	No value added beyond clinical judgment	12

^a^Data represent percentage of respondents out of ^b^ *n* = 38, ^c^ *n* = 51, ^d^ *n* = 91

### Treatment guidelines

A majority of respondents (53%) reported using a combination of resources to guide treatment decisions, most commonly EULAR/EUVAS recommendations (24%). A minority used site-specific standardized protocols (7%); others used pediatric textbook recommendations (4%), individualized approaches according to personal interpretation (4%), or advice from colleagues (6%). The majority of respondents (96%) believed in a need for pediatric treatment guidelines for GPA/MPA. Over half (58%) were interested in being involved in the process of consensus guideline development – 43% through an iterative survey – while others felt they did not have the time (13%) or relevant expertise (19%), or were unsure (9%). There was no association between group membership and the desire to participate in guideline development (OR = 1.4 [0.4, 5.1], *p* = 0.7206, *n* = 131), although respondents practicing outside the USA and Canada were more likely to want to participate than those within (OR = 4.2 [1.9, 9.0], *p* = 0.0011, *n* = 135). Most respondents supported consensus guidelines drafted by an expert group (69%) and believed in the need for a limited range of options to allow for comparative outcome assessment through a clinical registry (63%). Slightly less than half (46%) felt modification of recommendations for adult disease was an acceptable method for generating pediatric treatment guidelines.

### Treatment practices

All respondents followed a remission-induction and remission-maintenance model, switching from induction to maintenance therapy within 3-6 months. In adult studies, choice of induction agent may be adjusted based on measures of disease severity, primarily to limit use of aggressive life-saving treatment – specifically cyclophosphamide – that also has significant toxicity and may be unwarranted in milder disease. Two-thirds (67%) of respondents endorsed always using more aggressive treatment for patients with severe disease, while 32% reported only sometimes choosing induction therapy based on disease severity. Table 5 shows first-line induction and maintenance therapies preferred by respondents for treatment of AAV; respondents were not asked to distinguish between GPA- and MPA-specific regimens. In patients with severe disease not requiring intensive care, cyclophosphamide (CYC) was the first choice of remission-induction agent by 66% of respondents, while 31% stated they would choose rituximab (RTX). Use of RTX rather than CYC as an induction agent was more frequently reported by respondents from the USA (OR = 2.7 [1.3, 5.5], *p* = 0.0190, *n* = 139), those with greater than 5 years of experience (OR = 3.8 [1.3, 12.5], *p =* 0.0279*, n =* 137), and those who had seen more than 10 patients with GPA/MPA in their careers (OR = 4.39 [2.1, 9.1], *p =* 0.0011*, n =* 133).

**Table 5:** Induction and maintenance agents used by respondents^a^

Indication	Agent	Respondents (%)
Induction agent in severe disease^b^	Cyclophosphamide	66
	Rituximab	31
	Other	2
Induction agent if not using CYC or RTX (e.g. for less severe disease)^c^	Methotrexate	40
	Azathioprine	30
	Mycophenolate	25
	Other	4
Maintenance therapy^d^	Azathioprine	45
	Methotrexate	23
	Mycophenolate	18
	Rituximab	11
	Other	3

^a^Data represent percentage of respondents out of ^b^ *n* = 140, ^c^ *n* = 138, ^d^ *n* = 141

**Fig. 1:**
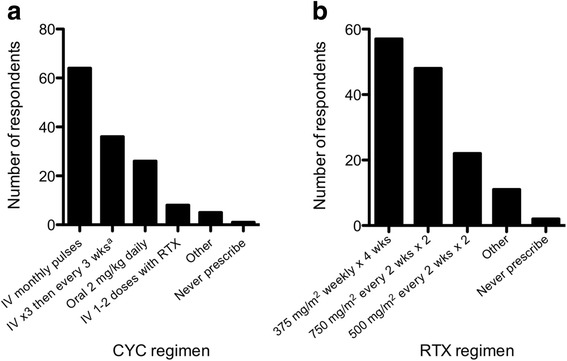
Typical dosing regimens prescribed for cyclophosphamide (CYC) and rituximab (RTX) induction therapy. Data represent number of respondents out of *n* = 140 for both agents. ^a^EUVAS protocol

Figure 1 shows the heterogeneity among prescribing practices for both CYC and RTX. Most respondents used CYC for induction until clinically inactive disease up to a maximum of 6 months (69%), while 16% treated for a defined duration between 3 and 7 months, 7% gave only 1-2 IV doses in conjunction with RTX, 5% continued induction therapy until clinical inactivity regardless of duration, and 2% employed some other regimen. The most common first choice of induction therapy in children not receiving CYC or RTX was methotrexate (MTX), followed by azathioprine (AZA) and mycophenolate mofetil (MMF) (Table 5).

The most commonly used remission-maintenance treatment was AZA, followed by MTX, MMF, and RTX (Table 5). There was no association between maintenance therapy and practice location (Canada vs. USA vs. other; *p* = 0.2009, *n* = 139), years of practice (<5 vs. 5 or more; *p* = 0.3042, *n* = 141), or total number of patients seen with GPA/MPA (<10 vs. 10 patients or more; *p* = 0.8328, *n* = 141). The provisional choice for maintenance therapy duration of 24 (46%), 18 (19%), 36 (14%), or 12 (13%) months was associated with total patient experience: 75% of respondents who had seen more than 10 GPA/MPA patients in their careers planned for at least 24 months of therapy, compared to 52% of those who had seen fewer than 10 patients (OR = 3.0 [1.4, 6.4], *p* = 0.0161, *n* = 127). Duration of maintenance therapy was not associated with practice location (Canada vs. USA vs. other; *p* = 0.3110, *n* = 130) or years of practice (<5 vs. 5 or more; OR = 1.6 [0.70, 3.89], *p* = 0.3109, *n* = 127). Concurrent corticosteroid duration initiated at induction was typically six (39%) or 12 (40%) months, with some respondents continuing for 18 (6%), 24 (4%), or 36 (0.7%) months and 11% chose variable periods according to disease severity. 93% of respondents routinely recommended plasma exchange in certain situations: severe pulmonary hemorrhage and/or rapidly progressive renal disease (76%), rapidly progressive renal disease only (8%), pulmonary hemorrhage only (5%), and with co-existent conditions (10%).

### Interest in clinical registries

All respondents felt that an international collaborative registry was important for comparative outcome assessment of treatment strategies for GPA/MPA, with 64% of respondents selecting “very important” (mode 5, median 5, IQR 4-5). Primary motivations for participation in clinical studies or collaborative registries included the potential to improve outcomes for children with AAV (91%), access to available tools and resources (58%), endorsement by a formal network of investigators (49%), potential publication authorship (46%), and association with specific research objectives (40%). Only 15% of respondents felt that a monetary stipend was a major motivation. Registry-associated resources believed to be of most value included an automated PVAS calculator (82%), an online algorithm to stage disease severity with links to corresponding treatment guidelines (68%), an automated PVDI calculator (66%), a classification tool based on entered patient data (54%), and a printable table to track patient data over multiple visits (54%). Most clinicians had resources available to assist with the use of registry data entry and clinical tools, including computer and internet access in the clinic (80% each) and a trainee or fellow (58%). Fewer had a research assistant (47%), support for review board applications (37%), technology support (33%), or a research nurse (23%). Major barriers to registry participation included insufficient support for data entry (59%), lack of time (43%), burden of ethics review board approval (32%), and lack of patients (24%).

## Discussion

This survey emphasizes the limited individual experience among clinicians caring for children with GPA and MPA and reiterates clinicians’ aspirations to study childhood-onset vasculitis independently from adult disease [3]. International collaborations within the last decade have facilitated the development of pediatric-specific classification criteria for GPA [9] and scoring tools for disease activity and damage [12, 15]. They have also evaluated the pediatric utility of adult patient algorithms for classifying MPA [16] and staging disease severity [17]. Moreover, the recent revision of the CHCC definitions is relevant to both adults and children [1]. These newly developed criteria, definitions, and clinical tools are essential for systematic evaluation of drug efficacy. However, the present survey reveals limited uptake by pediatric caregivers and identifies several challenges in the development, implementation, and evaluation of pediatric treatment guidelines.

### Distinguishing GPA from MPA

Most respondents believed that the distinction between GPA and MPA in patients with AAV was important for prognostication, consistent with meta-analyses of adult studies showing lower 5-year survival in MPA versus GPA [18] and of pediatric studies showing less frequent relapses [18] and more severe renal disease [16] in MPA. While respondents were familiar with both the ACR 1990 criteria for GPA [9] and its EULAR/PRINTO/PRES 2008 pediatric adaptation, neither organization includes criteria to define MPA [8, 19]. Not surprisingly, respondents often used multiple methods to differentiate (subclassify) GPA and MPA phenotypes, and 25% reported sometimes not differentiating. Frequently, ANCA specificity to PR3 or MPO was used “erroneously” as the primary differentiating criterion to define GPA and MPA, respectively. Adult studies suggest that genetics, pathophysiology, and clinical outcomes may be more strongly associated with antibody status than with the clinical syndromes [20]; although the populations defined by PR3 or MPO antibodies overlap with GPA and MPA, respectively, they are not the same. Indeed, most respondents were not aware of the recently revised CHCC 2012 definitions that do not incorporate ANCA specificity but arguably provide the clearest discriminating definitions of GPA and MPA [1]. Moreover, less than 20% of respondents were familiar with the European Medicines Agency (EMA) classification algorithm or its pediatric modification [21, 22]. However, this is a complicated tool primarily used in the research setting.

### Clinical scoring tools

Multiple national and international rheumatology organizations have published treatment guidelines for management of AAV in adults [23–31]. Many require formal clinical measurement tools to assess disease “severity” (to stratify appropriate therapy) and “activity” (to determine when to start, stop, or modify therapy). These standardized measures also guide entry of adult patients into research studies and will inevitably guide research eligibility in children. However, one third of respondents reported only sometimes tailoring induction therapy to a formalized disease severity scale, suggesting that other factors – likely including informal assessment, histopathology [32], and specific organ involvement – influence management decisions. In children, PVAS [12] may show better correlation with treatment decisions than the BVAS v.3 [33]; nevertheless, equal proportions of respondents used BVAS or its variants. Furthermore, evaluation of permanent damage from disease or treatment is required for outcome assessment and may allow prediction of mortality [34]. The PVDI is a relatively recent pediatric modification of the adult VDI [14] yet to be validated [15]; however, few respondents used either tool. Lack of knowledge or familiarity – as well as perceived lack of utility – may account for some lack of uptake. Successful implementation of practice guidelines requiring standardized assessment tools will depend on improved clinician engagement, clear clinical utility, and feasibility of use.

### Treatment practices

Consistent with guidelines for treatment of adults with AAV, all survey respondents employed a remission-induction and remission-maintenance model. Although 25% of respondents used EULAR/EUVAS recommendations to guide their treatment decisions, many employed a combination of resources. There was significant variation in medication and dosing regimens for childhood GPA/MPA, with variable use of evidence-based recommendations from existing adult guidelines.

### Potential engagement in comparative effectiveness research

An overwhelming majority of respondents believed in the need for pediatric-specific treatment guidelines and supported guideline development by expert group consensus. It is not simple to reconcile these aspirations with the limited uptake of recent pediatric-specific research and consensus reports, although the described lack of knowledge or familiarity – as well as unclear added benefit beyond current practice – likely contribute. In view of the high frequency of renal disease (75%) among pediatric AAV patients [16] and the existing high level of cross-specialty collaboration, future efforts towards developing and implementing guidelines for managing patients with AAV must further engage pediatric nephrologists.

A recent European initiative to develop standards of care for pediatric rheumatic diseases, SHARE (Single Hub and Access point for Paediatric Rheumatology in Europe) [35], will soon provide consensus-based guidelines for management of pediatric vasculitides, including GPA [36]. While many of these recommendations will be based on adult data, most survey respondents favored longer-term comparative outcome assessment of a range of possible consensus approaches. Given barriers to uptake of existing pediatric-specific tools – many of which will be part of standardized guidelines – incentives for implementation are critical. A registry that provides treatment guidelines endorsed by national/international rheumatology organizations, access to clinical tools with automated scoring calculators, and individual patient summary progress reports might further improve engagement and uptake.

Limitations of this study include a sample population that likely underestimates true variability in practice given the selection bias associated with elective survey completion [37] and response rates below 50% [38]. Participants were reached through formal organizations and therefore may represent a particularly informed group, leading to overestimation of the use of clinical tools and guidelines. Moreover, the survey included few respondents outside Europe and North America, and therefore is limited in generalizability particularly with respect to practice variation in Asian and African countries.

## Conclusions

Taken together, these data suggest a need for and interest in consensus treatment guidelines for pediatric GPA and MPA. They also point to potential challenges associated with guideline development and implementation. Understanding the barriers to uptake of existing classification criteria and formal assessment tools will inform efforts to improve standardization of classification and assessment to guide therapy. Physicians’ aspirations for pediatric-specific, evidence-based treatments may motivate uptake of guidelines that evolve from this needs assessment survey and, imminently, from SHARE. Consensus-derived guidelines that include a range of specific treatment options – if provided together with mechanisms for comparative effectiveness research through an international registry – may facilitate clinician engagement and ultimately lead to improved outcomes for children with AAV.

## Additional file

Additional file 1: Copy of Needs-assessment Survey for Treatment of pediatric ANCA-associated vasculitis. (PDF 110 kb)

## Abbreviations

AAV: Antineutrophil cytoplasmic antibody-associated vasculitis
ANCA: Antineutrophil cytoplasmic antibody
ARChiVe: A registry for childhood vasculitis
AZA: Azathioprine
BVAS: Birmingham vasculitis activity score
CAPRI: Canadian alliance of pediatric rheumatology investigators
CARRA: Childhood arthritis and rheumatology research alliance
CHCC: Chapel hill consensus conference
CYC: Cyclophosphamide
EGPA: Eosinophilic granulomatosis with polyangiitis
EMA: European medicines agency
EULAR: European league against rheumatism
EUVAS: European vasculitis study group
GPA: Granulomatosis with polyangiitis
IQR: Interquartile range
MMF: Mycophenolate mofetil
MPA: Microscopic polyangiitis
MTX: Methotrexate
PedVas: Pediatric vasculitis initiative
PRES: Pediatric Rheumatology European Society
PRINTO: Paediatric rheumatology international trials organisation
PVAS: Pediatric vasculitis activity score
PVDI: Pediatric vasculitis damage index
RTX: Rituximab
SHARE: Single hub and access point for Paediatric Rheumatology in Europe
VDI: Vasculitis damage index
WGET: Wegener’s granulomatosis etanercept trial
