# Supplementary material for: Clinical practice variation and need for pediatric-specific treatment guidelines among rheumatologists caring for children with ANCA-associated vasculitis: an international clinician survey
Source: Pediatr Rheumatol Online J. 2017 Aug 7;15:61. doi: 10.1186/s12969-017-0191-z (PMC5545848; doi:10.1186/s12969-017-0191-z)
Supplement: Additional file 1: — Copy of Needs-assessment Survey for Treatment of pediatric ANCA-associated vasculitis. (PDF 110 kb) [file 12969_2017_191_MOESM1_ESM.pdf]

2015 SURVEY TOWARDS DEVELOPING CONSENSUS TREATMENT PROTOCOLS (CTPs) FOR PEDIATRIC ANCA-ASSOCIATED VASCULITIS (AAV

Please complete the survey below.

Thank you!

A. INTRODUCTION

This survey focuses specifically on granulomatosis with polyangiitis (GPA, formerly Wegener granulomatosis) and microscopic polyangiitis (MPA) in children, and excludes eosinophilic granulomatosis with polyangiitis (EGPA, formerly Churg-Strauss syndrome).

A list of abbreviations for the international organizations and research consortiums referred to in the survey is provided immediately below. Those identified by asterisk have developed evidence- based guidance documents (references 1-9) for managing adult patients with AAV. References to these and other classification systems, clinical scoring tools etcetera are also provided and listed as a printable PDF at the end of this survey for your information. It is not necessary for you to read these documents to complete the survey.

Your responses to this survey are anonymous. Thank you for your important contribution.

The survey is sponsored through the Canadian Institutes of Health Research (http://www.cihr-irsc.gc.ca/, grant TR2-119188), and endorsed by the Childhood Arthritis and Rheumatology Research Alliance (CARRA).

Referenced organizations, including those with guidelines* for management of AAV in adults:

CARRA (Childhood Arthritis and Rheumatology Research Alliance)
PReS (Pediatric Rheumatology European Society)
EULAR (European League Against Rheumatism)
EUVAS* (European Vasculitis Study Group)
PRINTO (Paediatric Rheumatology International Trials Organisation)
BSR* (British Society of Rheumatologists)
ISN* (International Society of Nephrology)
CARI* (Caring for Australasians with Renal Impairment)
JCS* (Japanese Circulation Society)
WGET (Wegener Granulomatosis Etanercept Trial)
EMA (European Medicines Agency)

ABBREVIATIONS

ANCA (Antineutrophil cytoplasmic antibodies)
AAV (ANCA-associated vasculitis)
GPA (Granulomatosis with polyangiitis) - Previously Wegener Granulomatosis
MPA (Microscopic polyangiitis)
EGPA (Eosinophilic granulomatosis with polyangiitis)- Previously Churg-Strauss Syndrome
BVAS (Birmingham Vasculitis Activity Score)
PVAS (Pediatric Vasculitis Activity Score)
VDI (Vasculitis Damage Index)
PVDI (Pediatric Vasculitis Damage Index)
DEI (Disease Extent Index)

B. PRACTICE DESCRIPTION AND EXPERIENCE

1. Are you a member of any of these listed groups? Select all that apply.

- ☐ CARRA
- ☐ PRES-CARRA Vasculitis working party
- ☐ PRES
- ☐ CAPRI
- ☐ Other national/ international pediatric rheumatology research groups
- ☐ None of the above

Please describe other pediatric rheumatology research group ____________________________________

2. In which country do you practice?

- ◯ Info not available
- ◯ Canada
- ◯ United States
- ◯ United States Minor Outlying Islands
- ◯ Afghanistan
- ◯ Albania
- ◯ Algeria
- ◯ American Samoa
- ◯ Andorra
- ◯ Angola
- ◯ Anguilla
- ◯ Antarctica
- ◯ Antigua And Barbuda
- ◯ Argentina
- ◯ Armenia
- ◯ Aruba
- ◯ Australia
- ◯ Austria
- ◯ Azerbaijan
- ◯ Bahamas
- ◯ Bahrain
- ◯ Bangladesh
- ◯ Barbados
- ◯ Belarus
- ◯ Belgium
- ◯ Belize
- ◯ Benin
- ◯ Bermuda
- ◯ Bhutan
- ◯ Bolivia
- ◯ Bosnia And Herzegovina
- ◯ Botswana
- ◯ Bouvet Island
- ◯ Brazil
- ◯ British Indian Ocean Territory
- ◯ Brunei Darassalam
- ◯ Bulgaria
- ◯ Burkina Faso
- ◯ Burundi
- ◯ Cambodia
- ◯ Cameroon
- ◯ Cape Verde
- ◯ Cayman Islands Cayman Islands
- ◯ Central African Republic
- ◯ Chad
- ◯ Chile
- ◯ China
- ◯ Christmas Island
- ◯ Cocos (Keeling) Islands
- ◯ Colombia
- ◯ Comoros
- ◯ Congo
- ◯ The Democratic Republic Of The
- ◯ Cook Islands
- ◯ Costa Rica
- ◯ Cote D Ivoire
- ◯ Croatia
- ◯ Cuba
- ◯ Cyprus
- ◯ Czech Republic
- ◯ Denmark
- ◯ Djibouti
- ◯ Dominica
- ◯ Dominican Republic
- ◯ East Timor
- ◯ Ecuador
- ◯ Egypt
- ◯ El Salvador
- ◯ Equatorial Guinea
- ◯ Eritrea

◯ Estonia
◯ Ethiopia
◯ Falkland Islands (Malvinas)
◯ Faroe Islands
◯ Fiji
◯ Finland
◯ France
◯ French Guiana
◯ French Polynesia
◯ French Southern Territories
◯ Gabon
◯ Gambia
◯ Georgia
◯ Germany
◯ Ghana
◯ Gibraltar
◯ Greece
◯ Greenland
◯ Grenada
◯ Guadeloupe
◯ Guam
◯ Guatemala
◯ Guinea
◯ Guinea-Bissau
◯ Guyana
◯ Haiti
◯ Heard Island And Mcdonald Islands
◯ Holy See (Vatican City State)
◯ Honduras
◯ Hong Kong
◯ Hungary
◯ Iceland
◯ India
◯ Indonesia
◯ Islamic Republic Of
◯ Iraq
◯ Ireland
◯ Israel
◯ Italy
◯ Jamaica
◯ Japan
◯ Jordan
◯ Kazakstan
◯ Kenya
◯ Kiribati
◯ Republic Of
◯ Kuwait
◯ Kyrgyzstan
◯ Lao People s Democratic Republic
◯ Latvia
◯ Lebanon
◯ Lesotho
◯ Liberia
◯ Libyan Arab Jamahiriya
◯ Liechtenstein
◯ Lithuania
◯ Luxembourg
◯ Macau
◯ The Former Yugoslav Republic Of
◯ Madagascar
◯ Malawi
◯ Malaysia
◯ Maldives
◯ Mali
◯ Malta
◯ Marshall Islands
◯ Martinique
◯ Mauritania
◯ Mauritius
◯ Mayotte
◯ Mexico

◯ Federated States Of
◯ Republic Of
◯ Monaco
◯ Mongolia
◯ Montserrat
◯ Morocco
◯ Mozambique
◯ Myanmar
◯ Namibia
◯ Nauru
◯ Nepal
◯ Netherlands
◯ Netherlands Antilles
◯ New Caledonia
◯ New Zealand
◯ Nicaragua
◯ Niger
◯ Nigeria
◯ Niue
◯ Norfolk Island
◯ Northern Mariana Islands
◯ Norway
◯ Oman
◯ Pakistan
◯ Palau
◯ Palestinian Territory, Occupied
◯ Panama
◯ Papua New Guinea
◯ Paraguay
◯ Peru
◯ Philippines
◯ Pitcairn
◯ Poland
◯ Portugal
◯ Puerto Rico
◯ Qatar
◯ Reunion
◯ Romania
◯ Russian Federation
◯ Rwanda
◯ Saint Helena
◯ Saint Kitts And Nevis
◯ Saint Lucia
◯ Saint Pierre And Miqelon
◯ Saint Vincent And The Grenadines
◯ Samoa
◯ San Marino
◯ Sao Tome And Principe
◯ Saudi Arabia
◯ Senegal
◯ Seychelles
◯ Sierra Leone
◯ Singapore
◯ Slovakia
◯ Slovenia
◯ Solomon Islands
◯ Somalia
◯ South Africa
◯ South Georgia And The South Sandwich Islands
◯ Spain
◯ Sri Lanka
◯ Sudan
◯ Suriname
◯ Svalbard And Jan Mayen
◯ Swaziland
◯ Sweden
◯ Switzerland
◯ Syrian Arab Republic
◯ Province Of China
◯ Tajikistan
◯ United Republic Of

◯ Thailand
◯ Togo
◯ Tokelau
◯ Tonga
◯ Trinidad And Tobago
◯ Tunisia
◯ Turkey
◯ Turkmenistan
◯ Turks And Caicos Islands
◯ Tuvalu
◯ Uganda
◯ Ukraine
◯ United Arab Emirates
◯ United Kingdom
◯ Uruguay
◯ Uzbekistan
◯ Vanuatu
◯ Venezuela
◯ Viet Nam
◯ Virgin Islands, British
◯ Virgin Islands, U.S.
◯ Wallis And Futuna
◯ Western Sahara
◯ Yemen
◯ Yugoslavia
◯ Zambia
◯ Zimbabwe

3. Do you practice rheumatology and see patients < 18 years of age?

◯ Yes ◯ No

Thank you for your participation. Please note that this survey is intended only for clinicians who care for children.

The survey is now completed. Please scroll to the bottom of the survey and select SUBMIT.

4. For how many years have you practiced as a rheumatologist? (Do not include years in a formal training program.)

◯ < 5
◯ 5-10
◯ 10-20
◯ 20-30
◯ 30-40
◯ >40

5. Do you see patients with GPA or MPA?

◯ Yes, for diagnosis only
◯ Yes, for diagnosis and ongoing followup
◯ No

* Survey Ends Here- Thank you for your participation. Please scroll to the bottom of the survey and select SUBMIT.

6. In what practice setting do you see patients with GPA or MPA to provide rheumatology care?

◯ By myself in a solo practice
◯ By myself within a group practice (may share on-call care of each other's patients)
◯ In a group practice, sharing diagnostic and treatment decisions on all patients
◯ Other (specify below)

Other (please describe)

____

How many clinicians are part of your group?

◯ 1
◯ 2
◯ 3
◯ 4
◯ 5
◯ 6
◯ 7
◯ 8
◯ 9
◯ 10
◯ more than 10

7. For your GPA/MPA patients with renal disease who have also been assessed by a nephrologist, who is primarily responsible for treatment decisions?

- ◯ Me or my rheumatology group
- ◯ A nephrologist
- ◯ It varies from patient to patient (perhaps depending on who sees the patient first)
- ◯ Me or my rheumatology group collaboratively with the nephrologist in separate clinic settings
- ◯ Me or my rheumatology group collaboratively with the nephrologist in a combined renal/rheumatology clinic

8. For questions below, please provide the numbers of pediatric onset GPA or MPA patients that you have diagnosed independently or with shared group-practice responsibility (Best estimates)

Newly diagnosed in the past year

- ◯ 0
- ◯ 1
- ◯ 2
- ◯ 3
- ◯ 4
- ◯ 5
- ◯ 6
- ◯ 7
- ◯ 8
- ◯ 9
- ◯ 10
- ◯ More than 10

Newly diagnosed in the past 5 years

- ◯ 0
- ◯ 1
- ◯ 2
- ◯ 3
- ◯ 4
- ◯ 5
- ◯ 6
- ◯ 7
- ◯ 8
- ◯ 9
- ◯ 10
- ◯ More than 10

Total that you are following in your current practice

- ◯ 0
- ◯ 1
- ◯ 2
- ◯ 3
- ◯ 4
- ◯ 5
- ◯ 6
- ◯ 7
- ◯ 8
- ◯ 9
- ◯ 10
- ◯ More than 10

Total ever seen wherever you have practiced

- ◯ 0
- ◯ 1
- ◯ 2
- ◯ 3
- ◯ 4
- ◯ 5
- ◯ 6
- ◯ 7
- ◯ 8
- ◯ 9
- ◯ 10-20
- ◯ More than 20

10. If you have not cared for 2 or more patients with GPA and/or MPA during the past five years, you may choose not to continue the survey.

- ◯ I choose to finish the survey now
- ◯ I would like to continue

Thank you- this is the end of the survey. Please scroll to the bottom of the form and select SUBMIT.

C. CLASSIFICATION

For the purposes of this survey, "classification" might be considered the process by which we distinguish one type of vasculitis from another whereas diagnosis might be considered the process of distinguishing vasculitis from some other category of disease such as infection. There are no formal diagnostic criteria for adult or pediatric vasculitis.

1. With which of these classification criteria or disease definitions are you familiar? Select all that apply.

- ☐ ACR 1990 Criteria (10) Provides a framework for classifying vasculitis according to vessel size PLUS specific classification criteria Wegener Granulomatosis (GPA) (11) and some other types of Vasculitis
- ☐ CHCC 1994: Report of Chapel Hill Consensus Conference of disease definitions with clinical and pathological descriptions of the various types of vasculitis using the ACR 1990 framework (12).
- ☐ EULAR/PRINTO/PRES 2008 criteria: Pediatric adaptation of ACR classification criteria for GPA (13) and 3 other types of Vasculitis.
- ☐ EMA classification algorithm 2007 (14) or Pediatric adaptation, 2012 (15): European Medicines Agency (EMA) algorithm for uniquely classifying AAV subtypes and polyarteritis nodosa
- ☐ CHCC 2012 (16): International revision of 1994 report (above) updating the framework for classifying vasculitis, disease nomenclature, and disease definitions with incorporation of new knowledge most notably the presence or absence of ANCA
- ☐ NONE OF THE ABOVE

2. After diagnosing a patient as having an ANCA associated small vessel vasculitis (AAV), do you sub-classify or differentiate the patient as having GPA versus MPA

- ○ Always
- ○ Sometimes
- ○ Never

3A. Which of the listed criteria/definitions/tools do you use to subclassify patients with AAV as having GPA versus MPA? Select all that apply.

- ☐ ACR 1990 criteria
- ☐ EMA classification algorithm 2007 (or pediatric modification)
- ☐ EULAR/PRINTO/PRES 2008 criteria
- ☐ CHCC 1994 or 2012 disease definitions
- ☐ The presence of cANCA/PR3 versus pANCA/MPO
- ☐ Other formal classification criteria
- ☐ Other informal criteria or definitions (e.g. might include informal evaluation of clinical, laboratory and histopathological findings)

Please describe the informal classification criteria: __________________________________

Please describe the other formal classification criteria: __________________________________

3B. Please describe why you do not choose to subclassify AAV patients as GPA versus MPA. Select all that apply.

- ☐ Classification schemes or definitions for adult disease should not be used for childhood AAV (unless validated in a pediatric population).
- ☐ Subclassifying patients with AAV to GPA versus MPA would not change my management.
- ☐ Use of formal classification tools or criteria is inconvenient.
- ☐ I am not familiar with these disease classification criteria, schemes, or definitions
- ☐ Other

If you chose other, please specify: ____________________

4. Other than GPA versus MPA, for your patient with AAV do you further subclassify their disease in any of these listed ways? Select all that apply.

- ☐ I never differentiate or subclassify further
- ☐ cANCA/PR3 vs. pANCA/MPO
- ☐ Granulomatous vs. non granulomatous
- ☐ Renal vs. non renal
- ☐ Other formal or informal classification criteria

Please describe other formal or informal classification criteria: ____________________

5. Why do you sub-classify patients with AAV as GPA versus MPA, or in other ways? Select all that apply.

- ☐ Prognostication
- ☐ Influences treatment choice
- ☐ Specific diagnosis required for accessing specific treatments
- ☐ Participating in clinical studies, clinical trials or a registry
- ☐ Participating in biological discovery research
- ☐ Other

If you choose other, please describe: ____________________

	1 (Not Important)	2	3	4	5 (Very Important)
6. How important to research is formal subclassification of AAV (e.g. GPA vs. MPA)?	◯	◯	◯	◯	◯

D. TREATMENT GUIDELINES

A 'remission-induction' and 'remission-maintenance' model of therapy is recommended in most guidelines for treating adults with AAV. The choice of specific drugs for remission-induction in individual patients is arguably determined by the DISEASE SEVERITY. The subsequent duration of treatment before switching to remission-maintenance, requires assessment of DISEASE ACTIVITY to determine whether the patient has inactive disease.

1. Do you follow an induction/remission model of treatment for children with GPA/MPA (i.e. initial induction therapy switched to maintenance therapy within 3-6 months)?

◯ Yes ◯ No

2. In treating children with GPA/MPA, which of the following usually guide your treatment decisions? Select one of the following.

- ◯ EULAR/EUVAS recommendations for adult vasculitis (with pediatric modified dosing)
- ◯ Standardized treatment protocols developed by you or your practice group
- ◯ Guidelines of your national pediatric rheumatology professional association
- ◯ Other formal published "adult rheumatology" guidelines
- ◯ Adult rheumatology textbook recommendations
- ◯ Pediatric rheumatology textbook recommendations
- ◯ "Individualized" with advice from colleagues (local, national, international, bulletin board)
- ◯ "Individualised" according to my personal interpretation of the current literature.
- ◯ Combination of the above

Please specify the pediatric body: ____________________

Please specify the adult organisation: ____________________

Please specify the text book: ____________________

Please specify the text book: ____________________

Please specify: ______________________________

3. Do you think there is a need for treatment guidelines for pediatric GPA/MPA?

- ◯ No, adult guidelines are sufficient.
- ◯ No, I do not use published treatment guidelines.
- ◯ Yes, pediatric guidelines would be helpful.

4. Which of the following processes for generating treatment guidelines would be acceptable to you? Select all that would be acceptable.

- ☐ Based on modification of recommendations for adult disease
- ☐ Based on consensus of an 'expert' group that includes pediatric experts
- ☐ Based on iterative survey consensus in which I could participate
- ☐ Guidelines that provide a limited range of treatment options to allow for comparative outcome assessment through a clinical registry
- ☐ Other

Please specify: ______________________________

5. Would you like to be involved in the process of developing consensus treatment guidelines ?

- ◯ Yes - I have considerable expertise in the management of pediatric GPA/MPA.
- ◯ Yes - I have limited experience but would like to be involved.
- ◯ No - I don't have the expertise.
- ◯ No - I don't have time.
- ◯ Unsure

E. DISEASE SEVERITY

For the purpose of stratifying treatments, assessing DISEASE SEVERITY helps differentiate between disease that is imminently life- or organ-threatening, versus disease with minor or limited manifestations involving non-critical organs.

1. When initiating treatment for GPA/MPA and excluding patients with critical disease, in general - do you tailor therapy to use more aggressive treatment for patients with "severe" disease, and less toxic therapy for "mild" disease?

- ◯ Always
- ◯ Sometimes
- ◯ Never
- ◯ Unsure

2. You most likely assess DISEASE SEVERITY based on clinical judgment, but have you also used any of the listed 'formal' clinical assessment tools to stage disease severity? Select all that apply.

- ☐ EUVAS severity score (localized/early systemic/generalized/severe/refractory classification) (17)
- ☐ WGET severity score (limited/severe classification) (18)
- ☐ Five factor score (19)
- ☐ Disease Extent Index (20)
- ☐ Birmingham Vasculitis Activity Score (BVAS) (21, 22)
- ☐ Birmingham Vasculitis Activity Score for Wegeners (BVAS for WG) - with designation of critical organ involvement (23)
- ☐ Pediatric Vasculitis Activity Score (PVAS) (24)
- ☐ Other
- ☐ None of the above

Please specify: ______________________________

3. If or when you do not use formal disease severity assessment tools, please explain your rationale. Select all that apply.

- ☐ I use histopathological findings.
- ☐ Formal severity staging would not change my management.
- ☐ Use of formal tools would not add value beyond my clinical judgment.
- ☐ Use of formal severity staging tools is inconvenient.
- ☐ I am not familiar with these tools.
- ☐ Other

Please specify: __________________________________

F. DISEASE ACTIVITY

Assessing the presence, absence or level of inflammation (i.e. DISEASE ACTIVITY) helps characterize improvement, flare or remission, and ultimately helps guide therapy decisions.

1. Disease activity can be assessed 'informally' based on clinical judgment (e.g. examination, routine laboratory inflammatory markers). Have you ever used any of the listed 'formal' clinical assessment tools for patients in your clinic to assess disease activity? Select all that apply.

- ☐ I never 'formally' assess disease activity
- ☐ Birmingham Vasculitis Activity Score (BVAS)
- ☐ BVAS Version 3
- ☐ BVAS/Wegener granulomatosis (BVAS/WG)
- ☐ Pediatric vasculitis activity score (pVAS)
- ☐ Physician's global assessment (PGA) - marked on a 10 cm scale
- ☐ Other formal disease activity measurement tool

Please describe: __________________________________

2. When do you formally assess disease activity of children with GPA/MPA with any version of BVAS, PVAS, PGA or other formal clinical tool?

- ◯ At the time of diagnosis only
- ◯ At the time of diagnosis and all follow-up visits
- ◯ At the time of diagnosis and some other visits
- ◯ At prescribed times for patients enrolled in clinical trials or other research studies
- ◯ Other

Please specify: __________________________________

3. I do not use a formal method for scoring disease activity because (select all that apply):

- ☐ Tools for adult disease should not be used for childhood AAV (unless validated in a pediatric population).
- ☐ Formal disease activity scores would not change my management.
- ☐ Use of formal tools would not add value beyond my clinical judgment.
- ☐ Use of formal activity scoring tools is inconvenient.
- ☐ I am not familiar with these tools.

	1 (Not Important)	2	3	4	5 (Very Important)
4. How important to clinical management is formal assessment of disease activity in GPA and MPA?	◯	◯	◯	◯	◯

	1 (Not Important)	2	3	4	5 (Very Important)
5. How important to research is formal assessment of disease activity in GPA and MPA?	◯	◯	◯	◯	◯

G. DAMAGE ASSESSMENT

DAMAGE from the disease or its treatment, is typically considered irreversible and is unaffected by treatment of active vasculitis. In adults, it is used as one measure of outcome, severity, and a predictor of future damage.

1. Which of these tools for formal assessment of disease damage have you used for patients in your clinic or in a clinical trial setting? Select all that apply.

- ☐ Vasculitis Damage Index (VDI)
- ☐ Pediatric Vasculitis Damage Index (pVDI)
- ☐ AAV Index of Damage
- ☐ Combined Damage Assessment Index
- ☐ Other
- ☐ I never formally assess disease damage; I assess informally based on my clinical judgment

Please specify: ____________________________________

2. When do you formally assess disease damage in children with GPA/MPA using any of the above clinical tools?

- ◯ At the time of diagnosis only
- ◯ At the time of diagnosis and all follow-up visits
- ◯ At some follow-up visits
- ◯ At prescribed times for patients enrolled in clinical trials or other research studies
- ◯ Other, including varied combinations of above

Please specify: ____________________________________

3. I do not use a formal method for scoring disease damage because (select all that apply):

- ☐ Tools for adult disease should not be used for childhood AAV (unless validated in a pediatric population).
- ☐ Formal disease damage scores would not change my management.
- ☐ Use of formal tools would not add value beyond my clinical judgment.
- ☐ Use of formal damage scoring tools is inconvenient.
- ☐ I am not familiar with these tools.

H. SPECIFIC TREATMENTS

Aligned with adult treatment approaches, choice of specific treatments for children with GPA/MPA might be stratified according to disease severity, broadly distinguishing mild to moderate, moderate to severe, and critical disease requiring intensive care. Each treatment might be administered in a variety of regimens.

With the intent of developing a narrow range of "acceptable" consensus treatment protocols for long-term study, the extent of the variation in use of some specific treatments needs to be captured. What do you actually do?

1. Considering patients presenting with "severe" disease (but not requiring intensive care), what would be your first choice of a remission induction agent?

- ◯ Cyclophosphamide
- ◯ Rituximab
- ◯ Methotrexate
- ◯ Azathioprine
- ◯ Mycophenolate
- ◯ Leflunomide
- ◯ Other

Please describe: ____________________________________

2. When you use cyclophosphamide for treating GPA/MPA, how do you usually prescribe it?	◯ I never prescribe it<br>◯ Daily oral dosing 2 mg/kg/day (with daily IV equivalent in the intensive care setting)<br>◯ Intravenous infusions: 15mg/kg every 2 weeks for 3 doses and then 3 weekly (according to a EULAR/EUVAS adult protocol modified for pediatrics)<br>◯ Intravenous infusions: 0.5 - 1.0 g/m^2 monthly IV pulses (following following NIH SLE protocol)<br>◯ Intravenous infusions: 0.5 - 1.0 g/m^2 one or two doses ONLY in conjunction with Rituximab<br>◯ Other regimen
Please describe:	__________________________________
3. When you use intravenous or oral cyclophosphamide for remission induction therapy of GPA/MPA, what is your initial duration of therapy?	◯ Until clinically inactive, regardless of duration<br>◯ Until clinically inactive, or for 6 months duration (whichever is the shorter)<br>◯ For a defined duration in months. Please specify below.<br>◯ One or 2 IV doses ONLY in conjunction with Rituximab<br>◯ Other strategy
Define duration of therapy (in months):	◯ 1 month<br>◯ 2 months<br>◯ 3 months<br>◯ 4 months<br>◯ 5 months<br>◯ 6 months<br>◯ 7 months<br>◯ 8 months<br>◯ 9 months<br>◯ 10 months<br>◯ 11 months<br>◯ 12 months<br>◯ More than 12 months
Please describe:	__________________________________
4. When you use Rituximab for treating children with GPA/MPA at diagnosis, what dosing schedule do you usually use?	◯ I never prescribe it<br>◯ 375 mg/m^2/dose IV once weekly for 4 doses<br>◯ 500 mg/ m^2/dose for two doses two weeks apart<br>◯ 750 mg/ m^2/dose for two doses two weeks apart<br>◯ Other regimen
Please describe:	__________________________________
5. If you choose not to use cyclophosphamide or rituximab for your first line of treatment in a child with GPA/MPA (perhaps with less severe disease), what is your first choice of immunosuppressive treatment other than corticosteroids?	◯ Methotrexate<br>◯ Azathioprine<br>◯ Mycophenolate<br>◯ Leflunomide<br>◯ Other
Please describe:	__________________________________
6. If you treat a patient with GPA/MPA using the remission-induction and remission-maintenance approach, what is your preferred maintenance treatment?	◯ Methotrexate<br>◯ Azathioprine<br>◯ Mycophenolate<br>◯ Leflunomide<br>◯ Rituximab<br>◯ Other - please describe:
Please describe	__________________________________

7. If a patient is responsive to remission-induction treatment within a 4-6 month time frame, what would be your initial (provisional) plan for duration of subsequent maintenance therapy (not including corticosteroids)?

◯ 6 months
◯ 12 months
◯ 18 months
◯ 24 months
◯ 36 months
◯ Other - please specify:

Please describe ________________________________

8. If a patient is responsive to remission-induction treatment within a 4-6 month time frame, what would be your initial (provisional) plan for duration of corticosteroid therapy? (i.e. describe duration from onset of initial remission-induction therapy to complete cessation of prednisone)

◯ 6 months
◯ 12 months
◯ 18 months
◯ 24 months
◯ 36 months
◯ Other - please specify:

Please describe ________________________________

9. For which of the following do you (would you) routinely recommend plasma exchange ?

☐ Rapidly progressive renal disease
☐ Severe pulmonary hemorrhage
☐ Rapidly progressive renal disease PLUS severe pulmonary hemorrhage
☐ I do not usually recommend.
☐ Other

Please describe: ________________________________

I. REGISTRIES AND OTHER RESOURCES

Because of the rarity of GPA and MPA in children, it is unlikely that there will be clinical trials to provide timely evidence-based treatment guidelines. Comparative outcome assessment will best be enabled when there are a narrow range of well-defined treatment strategies.

	1 (Not Important)	2	3	4	5 (Very Important)
1. How important is an international collaborative registry to achieve this goal?	◯	◯	◯	◯	◯

2. Please select the top three items from the list based on how much they would motivate you to participate in/contribute to clinical studies or collaborative registries.

☐ Contribution to research that will improve outcomes for children with CPV
☐ Endorsed by my formal network of investigators formal network (e.g. CARRA, PReS, other)
☐ Associated with specific research objectives
☐ Access to tools/resources available to participating physicians
☐ Potential authorship on publications
☐ Monetary stipend
☐ Other

Please specify: ________________________________

3. If you are participating in (or were to participate in) a clinical registry for pediatric patients with GPA/MPA (or other types of vasculitis), which of the listed registry-associated resources would be of most value to you? Select up to a maximum of five.

- ☐ Disease classification tool based on patient data entered
- ☐ Automated pediatric vasculitis activity score (pVAS) calculator
- ☐ Automated pediatric vasculitis damage index (pVDI) calculator
- ☐ Online algorithm based using entered clinical data to stage disease severity at diagnosis with links to corresponding treatment guidelines
- ☐ Printable summary sheets of data entered for each visit
- ☐ Printable table to track an individual patient's data over multiple visits (e.g. pVAS scores, lab values)
- ☐ Summary of treatments and outcomes of similar patients entered to the registry
- ☐ Print-outs for patients and families with information on AAV, therapies, and outcomes
- ☐ A central website that links to pediatric vasculitis-related resources
- ☐ Links to relevant literature
- ☐ Patient-reported outcome tool (e.g. Child Health Assessment Questionnaire)
- ☐ Pediatric CPV-specific bulletin board / listserv
- ☐ Other

Please specify: __________________________________

4. Registries may include online tools to assist in your clinical management. Which of the following resources to assist in registry contribution/use are available at your site? Select all that apply.

- ☐ None
- ☐ Research assistant
- ☐ Research nurse
- ☐ Trainee/fellow who can contribute to research projects
- ☐ Information technology support
- ☐ Institutional review board application support
- ☐ Computer access in clinic
- ☐ Internet access in clinic

5. Please select the top TWO barriers to your participation in clinical registries.

- ☐ I do not think that such registries are useful.
- ☐ I do not have enough patients to make the effort worthwhile.
- ☐ I do not have enough time to participate.
- ☐ I do not have sufficient research support for data entry.
- ☐ Institutional review board approval is too burdensome.
- ☐ Other

Please specify: __________________________________

Please add any additional comments on this survey below. __________________________________

REFERENCES

[Attachment: "2015 Childhood AAV Clinician Survey_reference list.pdf"]

LINKS TO OPEN ACCESS ARTICLES AND SITES

ACR 1990 Criteria

EMA classification algorithm 2007

EULAR/PRINTO/PRES 2008 Criteria

CHCC 2012

EUVAS Severity Score

WGET severity score

Five Factor Score (FFS)

Disease Extent Index

Birmingham Vasculitis Activity Score (BVAS)

Birmingham Vasculitis Activity Score for Wegeners (BVAS for WG)

Pediatric Vasculitis Activity Score for Wegeners (PVAS)
